# A Practical Treatise on the Sexual Disorders of the Male and Female

**Published:** 1898-04

**Authors:** 


### Books and Magazines.

A Practical Treatise on the Sexual Disorders of the Male and Female. By Robert W. Taylor, A. M., M. D., Clinical Professor of Venereal Diseases at the College of Physicians and Surgeons (Columbia College), New York; Surgeon to Bellevue Hospital, and Consulting Surgeon to the City (Charity) Hospital, New York. With seventy-three illustrations and eight plates in color and monotone. Published by Lea Bros. & Co., New York and Philadelphia. 1897. Cloth, $3.

This work contains 451 pages, including index, and professes to bring the subject in all its bearings up to date, including the anatomy of the male organs of generation. It includes a chapter on sexual abnormalities, and quite a good chapter on impotence of the male, while, as usual, the female side of the question receives scant attention. The book is not superior, in our judgment, to the work of Wm. A. Hammond, published several years ago, and is inferior in many respects to the more recent work of Krafft-Ebing. On the whole, however, it would be of value to one who has not already a work especially devoted to this subject. The mechanical execution is faultless, and its greatest fault is its lack of detail. J.

